# Estimating critical values from electrocardiogram using a deep ordinal convolutional neural network

**DOI:** 10.1186/s12911-022-02035-w

**Published:** 2022-11-16

**Authors:** Guodong Wei, Xinxin Di, Wenrui Zhang, Shijia Geng, Deyun Zhang, Kai Wang, Zhaoji Fu, Shenda Hong

**Affiliations:** 1 HeartVoice Medical Technology, Hefei, 230027 China; 2 Department of Electrocardiogram, The First Affiliated Hospital of USTC, Division of Life Sciences and Medicine, University of Science and Technology of China, Hefei, Anhui 230001 China; 3 Department of Mathematics, National University of Singapore, Singapore, 119077 Singapore; 4 School of Management, University of Science and Technology of China, Hefei, 230026 China; 5 National Institute of Health Data Science, Peking University, Beijing, 100191 China; 6 Institute of Medical Technology, Health Science Center of Peking University, Beijing, 100191 China

**Keywords:** Critical value, Deep neural network, Ordinal classification, Electrocardiogram

## Abstract

**Background:**

Critical values are commonly used in clinical laboratory tests to define health-related conditions of varying degrees. Knowing the values, people can quickly become aware of health risks, and the health professionals can take immediate actions and save lives.

**Methods:**

In this paper, we propose a method that extends the concept of critical value to one of the most commonly used physiological signals in the clinical environment—Electrocardiogram (ECG). We first construct a mapping from common ECG diagnostic conclusions to critical values. After that, we build a 61-layer deep convolutional neural network named CardioV, which is characterized by an ordinal classifier.

**Results:**

We conduct experiments on a large public ECG dataset, and demonstrate that CardioV achieves a mean absolute error of 0.4984 and a ROC-AUC score of 0.8735. In addition, we find that the model performs better for extreme critical values and the younger age group, while gender does not affect the performance. The ablation study confirms that the ordinal classification mechanism suits for estimating the critical values which contain ranking information. Moreover, model interpretation techniques help us discover that CardioV focuses on the characteristic ECG locations during the critical value estimation process.

**Conclusions:**

As an ordinal classifier, CardioV performs well in estimating ECG critical values that can help people quickly identify different heart conditions. We obtain ROC-AUC scores above 0.8 for all four critical value categories, and find that the extreme values (0 (no risk) and 3 (high risk)) have better model performance than the other two (1 (low risk) and 2 (medium risk)). Results also show that gender does not affect the performance, and the older age group has worse performance than the younger age group. In addition, visualization techniques reveal that the model pays more attention to characteristic ECG locations.

## Background

Critical values, which are also known as panic values, tell “when to panic” over abnormal health-related conditions [1]. With a few numerical critical values that summarize and simplify the complex medical circumstances, health professionals can provide timely and effective responses. Also, people without any medical background can easily assess the health problems based on the values. Critical values are always defined with decision boundaries estimated from laboratory tests or vital signs [2–5]. For example, if a routine blood test shows a person’s serum potassium level is less than 3.0 mmol/L, it is a serious warning that the subject is at risk for hypokalemia and will need hospitalization, otherwise he or she might suffer from severe ventricular arrhythmia due to digoxin toxicity. Moreover, mortality risk scores in the Intensive Care Unit (ICU), such as APACHE III score [6] or SAPS II score [7], can also be viewed as an extended concept of critical values.

The above mentioned critical values require clinical laboratory tests or medical monitoring devices, which limits their usage in everyday life. In this paper, we aim to extend the concept of critical value to Electrocardiogram (ECG or EKG), which is one of the most commonly used non-invasive diagnostic or health management tools for heart-related problems. Compared with laboratory tests or simple vital signs, it is more difficult to build a critical value estimator for physiological signals, such as ECGs. Due to the high sampling frequencies, complex patterns, and long trends, traditional machine learning methods might not learn effectively from these complex signals.

Recently, deep neural networks (or deep learning methods) have achieved state-of-the-art performances in many areas such as speech recognition, computer vision, and natural language processing [8]. They also show great potentials on cardiovascular management [9, 10], disease detection [11–22], and biometric human identification [23, 24], and many other ECG analysis tasks [25–29]. However, there are no deep learning models designed for ECG critical value estimation so far. Unlike the above mentioned research, critical value estimation is neither a regression task nor a pure classification task. It is actually an ordinal classification task [30], which outputs categories that involve certain order relationships. As a result, the existing regression or classification models cannot be used directly to solve the ECG critical value estimation task.

In this paper, we present a method to estimate ECG critical values based deep learning techniques. We first propose a mapping from common ECG diagnostic conclusions (ECG statements) to ECG critical values. Then, we build an automatic critical value estimation model named CardioV, which is a 61-layer deep neural network based on neural architecture search and other advanced techniques in general Artificial Intelligence (AI) research areas [31–38]. Since the critical values have orders, we define the problem as a novel ordinal classification multi-task problem [30]. In order to obtain the probabilities of “no risk (critical value 0)”,“low risk (critical value 1)”, “medium risk (critical value 2)”, and “high risk(critical value 3)”, we train the model to learn the probabilities of being greater than “no risk ”, “low risk ” or “medium risk”, then convert them into probabilities of the four ordered critical values. We conduct experiments on a large public ECG dataset named PTB-XL [39]. The mean absolute error on the test set is 0.4984, and the average ROC-AUC score is 0.8735. Results also show that the agreement of model-cardiologist is comparable with that of cardiologist–cardiologist. In addition, our ablation study reveals that CardioV is better than the baseline deep learning models. With ECG critical values, people can easily assess their heart conditions and be aware of the critical situations.

## Methods

### Dataset

We use PTB-XL^1^ [39] from PhysioNet [40] to build and evaluate our method. The PTB-XL ECG dataset is a large publicly available ECG dataset, which contains 21,837 10-s clinical 12-lead ECG recordings from 18,885 patients (52% male and 48% female), ranging in age from 0 to 95. The waveform files are stored in WaveForm DataBase (WFDB) format with 16 bit precision at a resolution of 1 μ V/LSB and a sampling frequency of 500 Hz. A downsampled version of the waveform data with a sampling frequency of 100 Hz is released for the convenience of users. We use the 500 Hz ECG data in our experiments and preprocess the raw data using a bandpass filter of 0.5–50 Hz. The raw ECG data are annotated by up to two cardiologists, who assign potentially multiple ECG statements to each record [39]. There are 71 different ECG statements within five categories: Normal ECG (9528 records), Myocardial Infarction (5486 records), ST/T Change (5250 records), Conduction Disturbance (4907 records), and Hypertrophy (2655 records). The details of the 71 ECG statements can be found in our critical value mapping table (Table 2). The overall statistics is shown in Table 1.

**Table 1:** Overall statistics of PTB-XL dataset

Item	Statistics
# Records	21,837
Normal ECG	9528
Myocardial infarction	5486
ST/T change	5250
Conduction disturbance	4907
Hypertrophy	2655
Duration	10 s
Total patients	18,885
Gender	
Male	11,379 (52%)
Female	10,458 (48%)
Age	
Mean, (min, max)	60, (2, 95)
< 65, $\ge$ 65	12,331, 9417

### Mapping from ECG statements to critical values

We start with ECG statements conforming to the Standard Communication Protocol for Computer-assisted Electrocardiography (SCP-ECG) standard, which covers diagnostic, form, and rhythm statements. Based on the SCP-ECG standard and the 2017 Chinese expert consensus [41], we create the mapping between critical values and ECG statements as shown in Table 2. The resulting ECG critical values have four levels, which are 0 (No Risk), 1 (Low Risk), 2 (Medium Risk), and 3 (High Risk). Their ordinal relationships are as follow:

1 $$\begin{aligned} \begin{aligned} 0 (no\ risk)< 1 (low\ risk)< 2 (medium\ risk) < 3 (high\ risk) \end{aligned} \end{aligned}$$

**Table 2:** The mapping of ECG statements to critical values

Critical Values	Labels
0, No risk (9148 Records)	Normal ECG (NORM), non-specific T-wave changes (NT_), sinus rhythm (SR)
1, Low Risk (5322 Records)	Non-diagnostic T abnormalities (NDT), left anterior fascicular block (LAFB), incomplete right bundle branch block (IRBBB), non-specific ST changes (NST_), complete right bundle branch block (CRBBB), non-specific ischemic (ISC_), normal functioning artificial pacemaker (PACE), first degree AV block (1AVB), left atrial overload/enlargement (LAO/LAE), ischemic in lateral leads (ISCLA), incomplete left bundle branch block (ILBBB), right atrial overload/enlargement (RAO/RAE), left posterior fascicular block (LPFB), right ventricular hypertrophy (RVH), septal hypertrophy (SEHYP), sinus tachycardia (STACH), atrial premature complex (PAC), abnormal QRS (ABQRS), high QRS voltage (HVOLT), inverted T-waves (INVT), low amplitude T-waves (LOWT), prolonged PR interval (LPR), low QRS voltages in the frontal and horizontal leads (LVOLT), sinus arrhythmia (SARRH), sinus bradycardia (SBRAD), non-specific ST depression (STD_), non-specific ST elevation (STE_), T-wave abnormality (TAB_)
2, Medium Risk (3241 Records)	Left ventricular hypertrophy (LVH), complete left bundle branch block (CLBBB), ischemic in anterolateral leads (ISCAL), non-specific intraventricular conduction disturbance (block) (IVCD), ventricular premature complex (PVC), subendocardial injury in anteroseptal leads (INJAS), ischemic in anteroseptal leads (ISCAS), ischemic in inferior leads (ISCIN), ischemic in inferolateral leads (ISCIL), atrial flutter (AFLT), atrial fibrillation (AF), electrolytic disturbance or drug (former EDIS) (EL), paroxysmal supraventricular tachycardia (PSVT), digitalis-effect (DIG), ischemic in anterior leads (ISCAN), second degree AV block (2AVB), subendocardial injury in inferolateral leads (INJIL), subendocardial injury in lateral leads (INJLA), subendocardial injury in inferior leads (INJIN), bigeminal pattern (unknown origin, SV or Ventricular) (BIGU), premature complex(es) (PRC(S)), Q waves present (QWAVE), supraventricular arrhythmia (SVARR), supraventricular tachycardia (SVTAC), trigeminal pattern (unknown origin, SV or Ventricular) (TRIGU), voltage criteria (QRS) for left ventricular hypertrophy (VCLVH)
3, High Risk (4126 Records)	Inferior myocardial infarction (IMI), anteroseptal myocardial infarction (ASMI), inferolateral myocardial infarction (ILMI), anterior myocardial infarction (AMI), anterolateral myocardial infarction (ALMI), long QT-interval (LNGQT), Wolf-Parkinson-White syndrome (WPW), lateral myocardial infarction (LMI), inferoposterolateral myocardial infarction (IPLMI), subendocardial injury in anterolateral leads (INJAL), inferoposterior myocardial infarction (IPMI), posterior myocardial infarction (PMI), third degree AV block (3AVB), ST-T changes compatible with ventricular aneurysm (ANEUR)

### Deep neural network for modeling ECG

Deep learning methods especially convolutional neural networks (CNNs) have achieved state-of-the-art performances in ECG modeling [28]. We design our ECG classification CNN model with the neural architecture space searching technique that is adapted to find the best models for image classification [31]. The resulting network contains 61 layers which includes 7 stages of convolutional blocks connected with shortcut residual connection [32, 33], one global average pooling layer, and one fully connected dense layer. Each block consists of one convolutional layer with kernel size 1 (Conv1), one aggregated convolutional layer [34] with kernel size 16 and 16 groups (ConvK), and another convolutional layer with kernel size 1 (Conv1). Before each convolution layer, we apply batch normalization (BN) [35], Swish activation [36], and dropout (DO) [37]. We also introduce the channel-wise attention mechanism (SE block) [38] to improve the model performance. The first block of each stage downsample its input by a factor of 2, and the corresponding shortcut connections downsample the identity input using a max pooling operation by a factor of 2. The detailed model architecture is shown in Table 3.

**Table 3:** Model architecture

Stage	Layers	Output size
Input		(*, 12, 5000)
Stage 1	(Conv1, ConvK, Conv1) × 2	(*, 64, 2500)
Stage 2	(Conv1, ConvK, Conv1) × 2	(*, 160, 1250)
Stage 3	(Conv1, ConvK, Conv1) × 2	(*, 160, 625)
Stage 4	(Conv1, ConvK, Conv1) × 3	(*, 400, 312)
Stage 5	(Conv1, ConvK, Conv1) × 3	(*, 400, 156)
Stage 6	(Conv1, ConvK, Conv1) × 4	(*, 1024, 78)
Stage 7	(Conv1, ConvK, Conv1) × 4	(*, 1024, 39)
Pooling	Global Average	(*, 1024)
Prediction	Dense	(*, 3)

ECG length $n=5000$, number of leads $d=12$, and output dimension $c=3$. The first dimension $*$ represents number of samples in a batch

Formally, we use $\varvec{X} \in {\mathbb {R}}^{d \times n}$ to represent input ECG data, where *n* is the length of ECG, *d* is the number of leads which is 12 in our case. We also use ${\mathcal {F}}$ to represent our deep neural network. The predicted logits $\varvec{z} \in {\mathbb {R}}^{c}$ can then be represented as:

2 $$\begin{aligned} \begin{aligned} \varvec{z} = {\mathcal {F}}(\varvec{X}). \end{aligned} \end{aligned}$$

### Training via ordinal classification

For the ECG critical value estimation task, the common idea would be to build a deep model to implement a classification task. That is, given predicted logits $\varvec{z} \in {\mathbb {R}}^{c}$, for classification task we first apply softmax on $\varvec{z}$ to get probabilities $\varvec{p} \in [0,1]^{c}$, then optimize deep neural network ${\mathcal {F}}$ via cross-entropy loss. However, the classification task only distinguishes different classes, which does not model the ordinal relationship shown in Eq. 1.

**Fig. 1:**
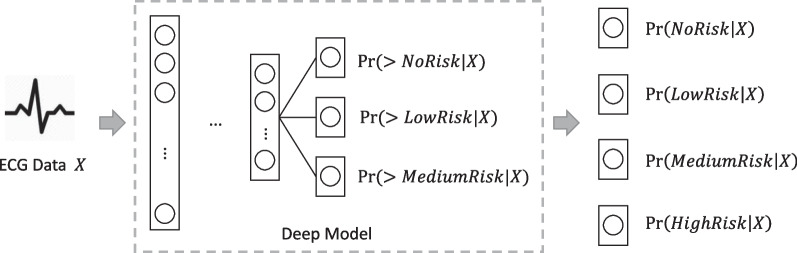
Framework

To solve this problem, we define the task as an ordinal classification task [30] rather than a simple classification task. The framework of our method is shown in Fig. 1. Ordinal classification task has the ability to learn from the ordinal relationship of classes. Intuitively, in our setting, the ordinal classification task can be regarded as a multi-task classification problem with the following three tasks:

- Task 1: whether the ECG critical value is higher than no risk? The probability is denoted as $Pr(>NoRisk\vert \varvec{X})$;
- Task 2: whether the ECG critical value is higher than low risk? The probability is denoted as $Pr(>LowRisk\vert \varvec{X})$;
- Task 3: whether the ECG critical value is higher than medium risk? The probability is denoted as $Pr(>MediumRisk\vert \varvec{X})$.

Formally, given predicted logits $\varvec{z} \in {\mathbb {R}}^{c}$, for ordinal classification task we first apply sigmoid on $\varvec{z}$ to get probabilities $\varvec{p} \in [0,1]^{c}$ (Eq. 3), then optimize objective *L* of deep neural network ${\mathcal {F}}$ via multi-task binary cross entropy (BCE) loss (Eqs. 4, 5). The label $\varvec{y}$ is computed based on Table 4. In addition, *c* is set to 3, $\varvec{p}[0]=Pr(>NoRisk\vert \varvec{X})$, $\varvec{p}[1]=Pr(>LowRisk\vert \varvec{X})$, and $\varvec{p}[2]=Pr(>MediumRisk\vert \varvec{X})$.

3 $$\begin{aligned} \varvec{p}= & {} sigmoid(\varvec{z}) \end{aligned}$$

4 $$\begin{aligned} L= & {} \frac{1}{c}\sum _{i=1}^{c}BCE(\varvec{p}[i], \varvec{y}[i]) \end{aligned}$$

5 $$\begin{aligned} BCE(p, y)= & {} y \cdot \log p + (1 - y) \cdot \log (1 - p) \end{aligned}$$

**Table 4:** Computing referenced probabilities from critical values

Critical value	$Pr(>NoRisk\vert \varvec{X})$	$Pr(>LowRisk\vert \varvec{X})$	$Pr(>MediumRisk\vert \varvec{X})$
0 (no risk)	0	0	0
1 (low risk)	1	0	0
2 (medium risk)	1	1	0
3 (high risk)	1	1	1

Then, we transform $Pr(>NoRisk\vert \varvec{X})$, $Pr(>LowRisk\vert \varvec{X})$ and $Pr(>MediumRisk\vert \varvec{X})$ into probability of each critical value: $Pr(NoRisk\vert \varvec{X})$, $Pr(LowRisk\vert \varvec{X})$, $Pr(MediumRisk\vert \varvec{X})$ and $Pr(HighRisk\vert \varvec{X})$ based on Eq. 6. The final output is the class with the highest probability.

6 $$\begin{aligned}&Pr(NoRisk\vert \varvec{X})= 1 - Pr(>NoRisk\vert \varvec{X}) \\ &Pr(LowRisk\vert \varvec{X})= Pr(NoRisk\vert \varvec{X}) - Pr(>LowRisk\vert \varvec{X}) \\ &Pr(MediumRisk\vert \varvec{X})= Pr(LowRisk\vert \varvec{X}) - Pr(>MediumRisk\vert \varvec{X}) \\ &Pr(HighRisk\vert \varvec{X})= Pr(>MediumRisk\vert \varvec{X}). \\ \end{aligned}$$

### Implementation details

We split the entire dataset by subject and obtain a training set with 17,741 samples (80% subjects), a validation set with 2193 samples (10% subjects), and a test set with 2203 samples (10% subjects). The model is built and trained with the PyTorch Python package. We choose Adam [42] optimizer with back-propagation, and add weight normalization to avoid overfitting. The batch size is set to be 256 samples, and the original learning rate is set to be 0.001. When the validation performance stops improving, we reduce the learning rate by a factor of 0.3. Compared with conventional classification, it is more difficult to train the ordinal multi-task classification. To solve the problem, we first train the model with conventional cross-entropy loss, and then conduct a finetuning after replacing the objective to ordinal loss. The results are reported on the test set.

### Evaluations

Our evaluation measurements include mean absolute error (MAE), receiver operating characteristic (ROC) curve of each class, area under the ROC curve (ROC-AUC, or just AUC) of each class, and the average value of AUC scores. The ROC curve is first computed based on the predicted probability and ground truth of each label directly without a predefined threshold, then defined as the curve of the true positive rate versus the false positive rate at various thresholds ranging from zero to one. We also ask the cardiologists to revise wrong predicted cases and calculate the agreement of model-cardiologist and the agreement of cardiologist-cardiologist. Moreover, we analyze $MAE=3$ cases, which represent serious errors in the model, one by one. Finally, in order to explain the model, we use the Grad-CAM [43] method to obtain the corresponding heat maps for the layers of interest. Through the heat maps, we can find the positions of the signal that the model is concerned about in the corresponding layers.

## Results

### Classification results

**Fig. 2:**
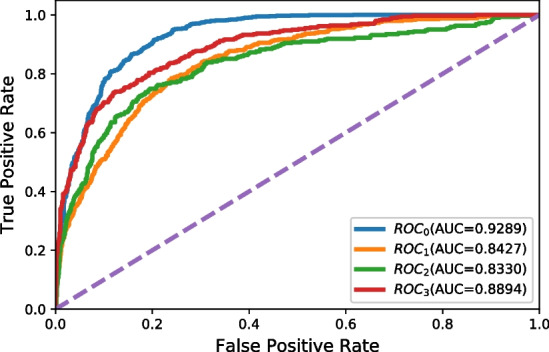
ROC curves of 4 classes of CardioV

The results of the ROC curves for each class are shown in Fig. 2. We can see that all four classes achieve higher than 0.8 ROC-AUC scores. We also observe that 0 (No Risk) and 3 (High Risk) are higher than the other two. The reason might be that the intermediate values (1 and 2) are more difficult to predict than the extreme values (0 and 3).

We then evaluate the model performance on different patient subtypes. We further divide the test set into subgroups by gender (male, female), and by age (age < 65, age $\ge$ 65), and show the results in Table 5. In terms of genders, the male and female groups have close performances on all evaluations, which indicates that the model is fair towards different genders. For ages, the age < 65 group is much better than the age $\ge$ 65 group. The reason might be that elders have age-related issues which could affect the heart but are difficult to be identified with ECG.

Moreover, we compare the agreement of model-cardiologist and the agreement of cardiologist-cardiologist. We first extract wrong predicted cases ($MAE \ge 1$), and then ask an individual cardiologist to revise these cases. After that, we analyze these results by comparing original labels (annotated by other cardiologists), model predictions, and revised labels. The total number of incorrectly predicted cases is 674. After revising, the cardiologist agrees with the original labels in 259 samples, agrees with model predictions in 361 samples, and disagrees with both in 54 samples. In this case, we can see that the agreement between model and cardiologist (model-cardiologist) is $361/674=53.56\%$, which is higher than the agreement between cardiologist and cardiologist (cardiologist-cardiologist) $259/674=38.43\%$. The disagreement of ECG diagnosis among cardiologists has already been discovered in previous research [25]. The result suggests that our method has at least comparable performance with cardiologists.

**Table 5:** Results of different subtypes

Subtype	MAE	$AUC_0$	$AUC_1$	$AUC_2$	$AUC_3$	*AUC*
Full	0.4934	0.9289	0.8421	0.8330	0.8894	0.8735
Male	0.4686	0.9304	0.8549	0.8254	0.9001	0.8777
Female	0.5197	0.9222	0.8298	0.8381	0.8754	0.8676
Age < 65	0.3882	0.8971	0.8725	0.8132	0.8975	0.8701
Age $\ge$ 65	0.6108	0.9324	0.7974	0.8214	0.8634	0.8537

### Ablation study

We compare CardioV with two ablation study baselines: classification and regression. We implement classification with the same model architecture, and replace our ordinal classification objective with the four-class cross-entropy objective. We also implement regression with the same model architecture, but optimize a mean squared error (MSE) objective to predict the numerical critical values. From the results shown in Table 6, we see that CardioV performs better than both classification and regression, which suggests that ordinal classification is a good choice when dealing with classification tasks with definite ordinal relationships among categories.

**Table 6:** Results of different methods

Method	MAE	$AUC_0$	$AUC_1$	$AUC_2$	$AUC_3$	*AUC*
CardioV	0.4934	0.9289	0.8421	0.8330	0.8894	0.8735
Classification	0.5247	0.9259	0.8233	0.8360	0.8838	0.8673
Regression	0.5811	–	–	–	–	–

### Case study

**Fig. 3:**
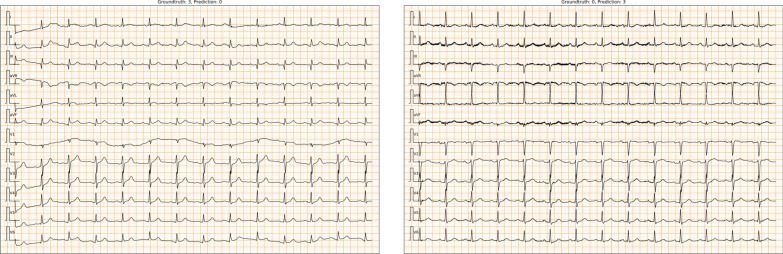
Distorted ECG cases. (Left) ECG with low-frequency baseline drift. (Right) ECG with high-frequency noise

**Fig. 4:**
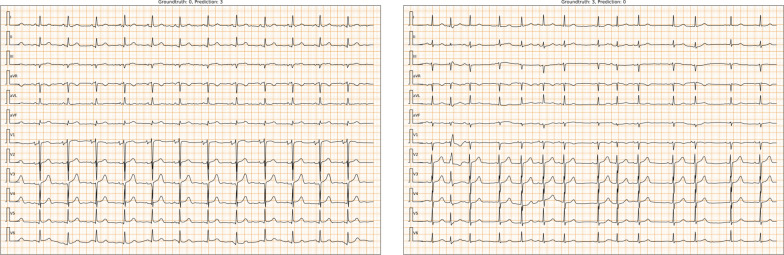
ECG cases that are difficult to be diagnosed. (Left) ECG with small R wave or pathological Q wave. (Right) ECG with frequent atrial premature complex

Finally, we examine the $MAE=3$ cases which might lead to serious consequences in the real-world applications. The total number of $MAE=3$ wrong predicted cases is 15. Among these cases, we find that 9 have distortions with low-frequency baseline drift (Fig. 3 Left) or high-frequency noise (Fig. 3 Right). The other cases are themselves difficult to be identified. For example, Fig. 4 (Left) shows the tiny R wave or pathological Q wave, which mainly exists in ECGs of people who have old inferior wall myocardial infarction (old IMI), but could also appear in ECGs of healthy people. Figure 4 (Right) shows an ECG with frequent atrial premature complex, which might be recognized as sinus arrhythmia.

**Fig. 5:**
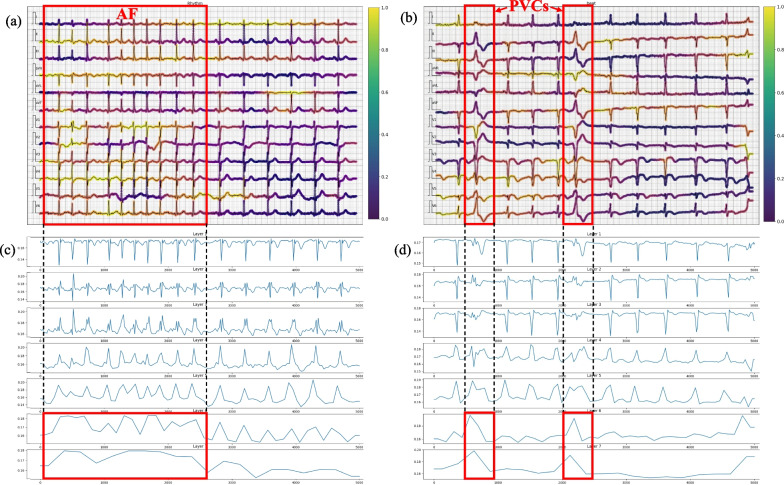
Visual interpretation of the model. **a**, **b** The ECGs of AF and PVCs, which are overlaid with the heat maps calculated by the Grad-CAM method. **c**, **d** The weights of heat maps for each stage corresponding to **a**, **b** (the highest stage is at the bottom). The red blocks mark the characteristic ECG locations

In addition, we apply gradient-weighted class activation mapping (Grad-CAM) to obtain the heat maps of the last convolutional layers for each stage to interpret the model. The highlighted areas represent the locations that the model focus on. To visualize them, we select two representative types of ECGs, which are ECGs of “rhythm-type” AF (characterized by an irregularly irregular rhythm) and ECGs of “beat-type” PVCs (characterized by wide QRS complexes). Figure 5a, b show the selected ECGs of AF and PVCs, which are overlaid with heat maps of the last convolution layer calculated by the Grad-CAM method. We see that most of the characteristic locations are brighter than other areas. To further explore the model’s hierarchical focus locations, we combine the heap map weights of all 12 leads for the last layer of each stage, and plot the weights from top to bottom in layer arrangement order (see Fig. 5c, d). The results show that the higher layers pay more attention to the characteristic ECG locations.

## Discussion

Critical value is a concept that is easy to understand, and even people without medical background can use it to identify different health conditions. The traditional critical values are associated with laboratory tests results and simple vital signs. However, in many cases, we need more complex signals to accurately assess the health conditions. As a physiological signal that can be collected easily and quickly, ECG is a good candidate to be mapped into critical values. Compared with laboratory tests and vital signs, ECG signals provide much more high-frequency information about the heart conditions, which also means it is difficult for traditional machine learning models to learn the features. In this paper, we propose a deep ordinal convolutional neural network named CardioV to automatically estimate the ECG critical value categories.

From the experimental results we find that the two extreme critical values (0 and 3) have better model performance than the two middle ones (1 and 2). The extreme critical values include normal and high risk conditions, which might be easier to predict than the other two, since the healthy state and severe condition could have more easily identifiable ECG characteristics. On the other hand, older people may have complex ECG signatures because their hearts may be affected by age-related diseases, so the model performs poorly in the older age group compared to the younger age group.

Since the main objective of this work is to demonstrate the feasibility of assessing critical values with ECGs, there are still several limitations. First, it does not combine other information of the patients, such as the blood routine test results. Second, without considering additional stratification rules, the same ECG recording can reflect a variety of heart diseases and can be subdivided into different critical grades. In the end, no specific suggestion of actions are associated with each critical value.

In the future, we plan to collect more data to enhance our model, and build a hierarchy of critical value estimator to support tiered medical services. Moreover, we would like to extend similar ideas to other physiological data, such as photoplethysmogram (PPG), electroencephalogram (EEG), and electromyogram (EMG), so people can easily understand these complex signals and take quick actions in life-threatening situations.

## Conclusion

In our study, we propose CardioV, an ordinal classifier, to estimate ECG critical value categories that can help people quickly identify different heart health conditions. Test results show that the model performs well in all four critical value categories. Furthermore, we observe three phenomena: extreme values (0 and 3) have better model performance than the other two; gender does not affect the performance; the older age group has worse performance than the younger age group. We also find that the agreement of model-cardiologist is comparable with that of cardiologist-cardiologist. The ablation study reveals that CardioV outperforms baseline deep learning models and validates that ordinal classification is suitable for identifying categories with ranking information. In addition, we interpret our model through activation visualization techniques, and discover that the model pays more attention to characteristic ECG locations, whether in “rhythm-type” or “beat-type” arrhythmia.

## Data Availability

The PTB-XL ECG dataset used in this study is available on the PhysioNet website https://physionet.org/content/ptb-xl/1.0.1/. The code of CardioV can be downloaded from the github repository: https://github.com/hsd1503/CardioV. We also deploy our trained model and create an online application: https://www.heartvoice.com.cn/criticalvalue/index_en. With the application, users can test the critical value analysis function using their own data or the provided sample data.

## Footnotes

1 https://physionet.org/content/ptb-xl/1.0.1/.
